# The induction of neuronal death by up-regulated microglial cathepsin H in LPS-induced neuroinflammation

**DOI:** 10.1186/s12974-015-0268-x

**Published:** 2015-03-19

**Authors:** Kai Fan, Daobo Li, Yanli Zhang, Chao Han, Junjie Liang, Changyi Hou, Hongliang Xiao, Kazuhiro Ikenaka, Jianmei Ma

**Affiliations:** Department of Anatomy, Dalian Medical University, West Section No. 9, South Road, Lvshun, Dalian, 116044, Liaoning China; Clinical Medicine of Seven-year Education, Dalian Medical University, Dalian, 116044, Liaoning China; Regenerative Medicine Center, the First Affiliated Hospital, Dalian Medical University, Dalian, 116011, Liaoning China; Graduate School, Dalian Medical University, Dalian, 116044, Liaoning China; Division of Neurobiology and Bioinformatics, National Institute for Physiological Sciences, Okazaki, 444-8787, Aichi Japan

**Keywords:** Cathepsin H, Microglia, Cytokines, Inflammation, Apoptosis

## Abstract

**Background:**

Neuroinflammation is a hallmark that leads to selective neuronal loss and/or dysfunction in neurodegenerative disorders. Microglia-derived lysosomal cathepsins are increasingly recognized as important inflammatory mediators to trigger signaling pathways that aggravate neuroinflammation. However, cathepsin H (Cat H), a cysteine protease, has been far less studied in neuroinflammation, compared to cathepsins B, D, L, and S. The expression patterns and functional roles of Cat H in the brain in neuroinflammation remain unknown.

**Methods:**

C57BL/6J mice were intraperitoneally injected with either 0.9% saline or lipopolysaccharide (LPS, 5 mg/kg). Immunohistochemistry (IHC) and *in situ* hybridization (ISH) were used to analyze expression and localization of Cat H in the brain. Nitrite assay was used to examine microglial activation *in vitro*; ELISA was used to determine the release of Cat H and proinflammatory cytokines (TNF-α, IL-1β, IL-6, IFN-γ). Cat H activity was analyzed by cellular Cat H assay kit. Flow cytometry and *in situ* cell death detection were used to investigate neuronal death. Data were evaluated for statistical significance with one-way ANOVA and *t* test.

**Results:**

Cat H mRNA was only present in perivascular microglia and non-parenchymal sites under normal conditions. After LPS injection, Cat H mRNA expression in activated microglia in different brain regions was increased. Twenty-four hours after LPS injection, Cat H mRNA expression was maximal in SNr; 72 h later, it peaked in cerebral cortex and hippocampus then decreased and maintained at a low level. The expression of Cat H protein exhibited the similar alterations after LPS injection. *In vitro*, inflammatory stimulation (LPS, TNF-α, IL-1β, IL-6, and IFN-γ) increased the release and activity of Cat H in microglia. Conversely, addition of Cat H to microglia promoted the production and release of NO, IL-1β, and IFN-γ which could be prevented by neutralizing antibody. Further, addition of Cat H to Neuro2a cells induced neuronal death.

**Conclusions:**

Taken together, these data indicate that the up-regulated microglial Cat H expression, release, and activity in the brain lead to neuronal death in neuroinflammation. The functional link of Cat H with microglial activation might contribute to the initiation and maintenance of microglia-driven chronic neuroinflammation.

**Electronic supplementary material:**

The online version of this article (doi:10.1186/s12974-015-0268-x) contains supplementary material, which is available to authorized users.

## Background

The central nervous system (CNS) is regarded as an immune-privileged site because of the blood-brain barrier (BBB) and an immunosuppressive environment. However, in many neurological conditions, there is a loss of immune privilege, for instance, in a variety of neurodegenerative diseases, including Alzheimer’s disease, Parkinson’s disease, Huntington’s disease, and amyotrophic lateral sclerosis [1-4]. In these diseases, certain microenvironmental factors, such as glia overactivation, cytokines overproduction, BBB permeability increase, and peripheral immune cells infiltration, are recognized to be one of prominent pathological features. These characteristic pathological changes are generally described as neuroinflammation, which are thought to contribute to neuronal injuries and aggravation of neurodegenerative diseases [5-7]. To date, much attention has been given to microglia, a pivotal cellular player in neuroinflammation [8-12].

Microglia are the immune cells resident in the CNS, serving the role of tissue maintenance and immune surveillance of the brain [13,14]. Under inflammatory conditions, microglia are over-activated and secrete various inflammatory mediators including nitric oxide (NO), reactive oxygen species (ROS), and proinflammatory cytokines such as TNF-α and IL-1β [15-17].

There is increasing evidence that activated microglia also induce the synthesis and secretion of lysosomal cathepsins [18-22], in particular at the early stage of inflammation, to trigger signaling pathways in a pathological cascade that aggravate neuroinflammation. Cathepsins, a large group of lysosomal proteases, are subdivided into three subgroups according to the amino acids of their active sites that confer catalytic activity: cysteine (cathepsins B, C, F, H, K, L, N, O, S, T, U, W, and X), aspartyl (cathepsins D and E), and serine cathepsins (cathepsins A and G) [23]. These proteases play a central role in a number of important cellular processes such as degradation of intracellular proteins, extracellular matrix remodeling, and apoptosis. To date, in neuroinflammation, most of the research has focused on cathepsins B, D, L, and S, which appear to be most active in inflammatory response and play a key role in the neurotoxic effects [24-28]. In contrast, cathepsin H (Cat H) has been far less studied in this aspect.

Cat H is a cysteine protease involved in lysosomal protein degradation [29-31]. Cat H is synthesized as an enzymatically inactive proenzyme (procathepsin H, 41 kDa) in endoplasmic reticulum and then proteolytically processed to an active single-chain, mature form (28 kDa) within the endosomes/lysosomes. Unlike other members of the family, Cat H is unique in that it is both an exopeptidase (aminopeptidase) and an endopeptidase with a pH optimum of about 6.5 to 6.8 [32-35]. Cat H acts as an important contributor to inflammatory pathologies and tumor metastasis depending largely on its aminopeptidase activity which is determined by residual octapeptide EPQNCSAT of the propeptide termed mini-chain that limits the access of substrates to the catalytic center [36-39]. It has been implied that Cat H provokes acute inflammation characterized by the accumulation of polymorphonuclear leukocytes (PMN) when injected intracutaneously into newborn rats [40]. Additionally, Cat H level was elevated in inflammatory acute lung injuries [41,42], pancreatitis [43], inflammatory myopathy [44], and atherogenesis [45]. Moreover, Cat H level has a significant positive correlation with the severity of inflammation in these diseases.

Despite the extensive literature describing Cat H and peripheral inflammatory diseases, there is limited insight into the CNS inflammatory processes that Cat H may regulate. There are several findings that Cat H immunoreactivity in the hippocampus is increased in animal model of cerebral ischemia, and Cat H activity increases in affected brain areas in Huntington’s disease [29]. These findings imply a potential role of Cat H in the neuroinflammatory pathogenesis of neurological diseases.

In the present study, we set out to gain further insight into the functional roles of Cat H in neuroinflammation by investigating the temporal and spatial expression patterns and cellular localization of Cat H in the brain in the lipopolysaccharide (LPS)-induced neuroinflammation, as well as impact of Cat H on microglial activation and of neuronal survival.

## Materials and methods

### Animals

Eight-week-old C57BL/6J mice weighing 25 to 30 g were used in the experiments. Animals were housed in groups of five per cage in a 22°C ± 2°C and 45% ~ 65% relative humidity environment under a normal light cycle room (12-h light/12-h dark; 8:00 a.m. light on ~ 8:00 p.m. light off). All animals had free access to food and water. All procedures were in accordance with the Dalian Medical University Guidelines for the proper care and use of Laboratory Animals and were approved by the Laboratory Animal Care and Use Committee of Dalian Medical University.

#### LPS treatment

Lipopolysaccharide (LPS, *Escherichia coli*, serotype O55:B5, Sigma-Aldrich, St. Louis, MO, USA) was used to induce an inflammatory response. LPS was injected intraperitoneally at a dose of 5 mg/kg dissolved in sterile, endotoxin-free 0.9% saline vehicle. Control injections were equivolume vehicle. The dosage of LPS was based on a previous study of LPS-induced neurotoxicity [46,47].

#### Tissue preparation

At the time points of 6 h, 24 h, 72 h, 7 days, and 10 days after LPS injection, mice were anesthetized with diethylether and perfused with 4% paraformaldehyde solution. The brains were removed and post-fixed and then cryoprotected in 20% sucrose solution and embedded in OCT compound (McCormick Scientific, St. Louis MO, USA); and serial 18 μm sagittal sections were made with a cryostat (Leica CM 3050S, Solms, Germany) and used for the immunohistochemical (IHC) and *in situ* hybridization (ISH) studies.

For ELISA, mice were perfused with ice-cold PBS, and then the brains were removed and homogenized on ice. The homogenates were centrifugated at 12,000 g for 5 min at 4°C. The supernate was stored at −80°C.

#### In situ *hybridization*

ISH was performed as described previously [48]. After hybridization of Cat H (NM_007801, 35 to 1,200 bp) cRNA probe, samples were incubated overnight with alkaline phosphatase-conjugated anti-digoxigenin (DIG) antibody (Roche, Basel, Switzerland) at 4°C. Color development was achieved by incubation with NBT/BCIP for 16 h at room temperature. Some sections were counterstained with Nuclear Fast Red for observation and analysis; others were processed for Iba-1, GFAP, and NeuN IHC staining after ISH, respectively. Images were captured using the Nikon digital camera system (DS-Fi1) in combination with microscopy (Nikon Eclipse 80i, Nikon, Tokyo, Japan). The number of cells expressing Cat H mRNA was counted with ImageJ 1.41 (National Institutes of Health, Bethesda, Maryland). Three sections from each mouse (with five mice per condition) were used for analysis.

#### Immunohistochemical staining

IHC was performed as described by Ma *et al.* [49]. The following antibodies were used: mouse anti-NeuN monoclonal antibody (1:1,000, Chemicon, EMD Millipore, Billerica, MA, USA), rabbit anti-GFAP polyclonal antibody (1:1,000, Dako, Glostrup, Denmark), rabbit anti-Iba1 polyclonal antibody (1:500, Wako, Osaka, Japan). Secondary antibodies were labeled with biotin (1:200, Vector Laboratory, Burlingame, CA, USA). After IHC reaction, images were captured using the Nikon digital camera system (DS-Fi1) in combination with microscopy (Nikon Eclipse 80i).

#### Real-time quantitative PCR

Total RNA was extracted from the brain using TRIzol reagent (Invitrogen, Carlsbad, CA, USA) according to the manufacturer’s protocol. Reverse transcription was performed using SuperScript II Reverse Transcriptase (Invitrogen). Real-time quantitative PCR was performed in 25 μL reaction volume using SYBR green PCR Master Mix (Thermo Scientific, Waltham, MA, USA), as described by the manufacturer. The primer sequences are the following: Cat H: forward: 5′ GAGCAGCAGCTGGTGGATTG 3′, reverse: 5′ CCATGATGCCCTTGTTGTATAGGA 3′; β-actin: forward: 5′ ATCATGTTTGAGACCTTCAACA 3′, reverse: 5′ CATCTCCTGCTCGAAGTCTA 3′. All primers were synthesized by Takara Biotechnology Co. Ltd. (Dalian, China). The thermal cycling conditions included denaturation step at 95°C for 10 min, followed by 40 cycles at 95°C for 15 s, 60°C for 30 s, and 72°C for 30 s and the final melting curve program with ramping rate of 0.5°C/s from 55°C to 95°C. Amplification specificity of PCR products was confirmed by melting curve analysis and agarose gel electrophoresis. Fold regulation values were calculated relative to the expression mean of the group displaying the lowest expression.

#### Cell culture

Primary microglia were harvested from primary mixed glial cell cultures prepared from neonatal C57BL/6J mice pups as previously reported (Fan *et al*., [18]). In brief, after the meninges were carefully removed, the neonatal brain was dissociated by pipetting. The cell suspension was plated in 10-cm culture dish at a density of one brain per two dishes in 10 mL DMEM (Sigma-Aldrich) containing 10% fetal bovine serum (FBS) (ICN Biomedicals, Santa Ana, CA, USA). After 14 to 21 days *in vitro*, mixed glia cultures were dissociated by trypsinization, and the cell suspension was seeded on a petri dish and incubated for 30 min in a CO_2_ incubator. Adherent cells were harvested as primary microglia. Microglia were reseeded in culture plates. The purity of microglia was approximately 99% as determined by CD11b (rat monoclonal IgG, clone M1/70, Abcam, Cambridge, UK) staining.

The immortalized murine microglial cell line BV2 was a kind gift from Dr. XF Wu (Dalian Medical University, China) and were maintained in DMEM supplemented with 10% FBS, 2 mM glutamine, and 100 U/mL penicillin/streptomycin at 37°C in 5% CO_2_ in a humidified atmosphere.

Prior to inflammatory treatments, primary microglia and BV2 cells were cultured in media without FBS for 24 h at 1.0 × 10^5^ cells/24-well plate.

#### Cell treatment

BV2 cells were incubated for 24 h with LPS (10 ng/mL), then the culture media were collected, centrifuged to remove any cellular material.

Cat H antibody (N-18, Santa Cruz Biotech, Dallas, TX, USA) was added to the Cat H solution or microglial conditioned media and incubated for 1 h to allow sufficient time for antibody binding, then the samples were transferred to BV2 cells and incubated for 24 h. Controls were carried out in which Cat H antibody was directly added to BV2 cells to investigate non-specific effects.

#### Measurement of nitrite content

The production of nitric oxide (NO) *in vitro* was evaluated indirectly by measuring nitrite concentration (KeyGEN biotechnology, Nanjing, China). Test samples were the media obtained from BV2 cells and primary microglia following treatment with LPS (10 ng/mL), TNF-α (1 ng/mL), IL-1β (1 ng/mL), IL-6 (1 ng/mL), or IFN-γ (50 ng/mL) for 24 h, respectively. The absorbance at the wavelength 540 nm was determined using a microplate reader (iMark, Bio-rad, Hercules, CA, Japan).

#### ELISA

Cat H and proinflammation factors levels in the conditioned media were determined by enzyme-linked immunosorbent assay (ELISA).

Test samples for Cat H include brain supernate obtained from LPS-injected mice, the media from primary microglia and BV2 cells activated by LPS (10, 100, 1,000 ng/mL), IL-1β, IL-6, TNF-α (1, 10, and 100 ng/mL in each case), IFN-γ (50,500,5,000 ng/mL) for 24 h, respectively, and the media from BV2 cells treated with Cat H (2 ng/μL) in combination with Cat H antibody. Test samples for proinflammation factors were media from BV2 cells exposed to recombinant active Cat H protein (Abcam) in 0.2, 2, and 20 ng/μL, respectively.

The assay was performed according to Cat H ELISA Kit protocols (R&D) and TNF-α, IL-1β, IL-6, and IFN-γ ELISA Kit protocols (Peprotech, Rehovot, Israel). A microplate reader (iMark, Bio-rad, Japan) was used to measure absorbance at 450 nm. Cat H concentration was expressed in U/g of total protein and proinflammation factors in pg/μg of total protein.

#### Cat H enzymatic activity assay

Cat H aminopeptidase activity was determined by degradation of synthetic fluorogenic substrate L-Arg-7-amido-4-methylcoumarin (L-Arg-AMC). Test samples are the conditioned media obtained from primary microglia and BV2 cells following treatments with LPS (10 ng/mL), IL-1β, IL-6, TNF-α (10 ng/mL in each case), and IFN-γ (500 ng/mL) for 24 h, respectively. The procedures were performed according to Cat H assay kit protocols (Genmed Scientifics Inc., Wilmington, DE, USA). All samples including control groups (50 μg Cat H protein/well in 96-well plate) were incubated with the L-Arg-AMC at 37°C for 60 min. The release of fluorescent AMC was measured at an excitation and emission wavelength of 360 and 460 nm, respectively, in EnSpire Multimode Plate Reader (PerkinElmer, Waltham, MA, USA). The fluorescent signal was calibrated using known concentrations of AMC. Data were presented as relative folds to untreated control.

#### Flow cytometry

Flow cytometry was used to evaluate neuronal death following addition of recombinant active Cat H protein (Abcam) in 2 and 20 ng/μL to the culture media of the neuroblastoma cell line Neuro2a cells, respectively. After incubation for 24 h, the cells were harvested by centrifugation at 2,000 rpm for 5 min. After washing twice with pre-cold phosphate-buffered saline (PBS, pH 7.4), cells were resuspended in binding buffer and then stained by Annexin V-FITC and propidium iodine (PI) for 5 to 15 min at room temperature. At last, these cells were subjected to flow cytometer (Becton Dickinson, Laguna Hills, CA, USA).

#### In situ *cell death detection*

Adherent Neuro2a cells on coverslips were incubated with recombinant active Cat H protein (Abcam) in 20 ng/μL for 24 h. *In situ* cell death detection kit (TMR red, Roche) was applied to perform TdT-mediated dUTP nick end labeling (TUNEL) staining to evaluate neuronal apoptosis. Briefly, the cells were fixed by 4% paraformaldehyde in PBS (pH 7.4) for 1 h at 4°C, washed in PBS, and then incubated in permeabilization solution (0.1% Triton X-100 in 0.1% sodium citrate) for 2 min at 4°C. Later, the cells were incubated in TUNEL reaction mixture for 1 h at 37°C in the dark. Hoechst 33342 was used for the nuclear counterstaining. Negative control cells were used as label solution instead of TUNEL reaction mixture. Positive control cells were incubated with DNase I recombinant (Takara) for 10 min at room temperature to induce DNA strand breaks, prior to labeling procedures. The numbers of apoptotic cells were counted using an Olympus IX70 inverted fluorescence microscope. Cells were scored in eight randomly chosen fields under a magnification of × 400 per coverslip on at least two coverslips per treatment. The percentage of cells positive for apoptosis was calculated.

#### Statistical analysis

Data were expressed as mean + standard error of the mean (SEM) from three independent experiments. All statistical analyses were performed using the Statistical Package for Social Sciences (Version 17.0). One-way ANOVA test was used to detect significance of the differences among more than two arithmetic means, followed by *post hoc* Scheffe test to detect the differences between two means. Student’s *t* test was used to detect the significance of differences between two means. *P* < 0.05 was considered statistically significant.

## Results

### Cat H expression was increased in the brain microglia in neuroinflammation evoked by LPS systemic injection

Previous studies found that the expression and enzymatic activity of Cat H was up-regulated in peripheral inflammation. However, the alterations of Cat H in neuroinflammation remain unknown.

**Figure 1:**
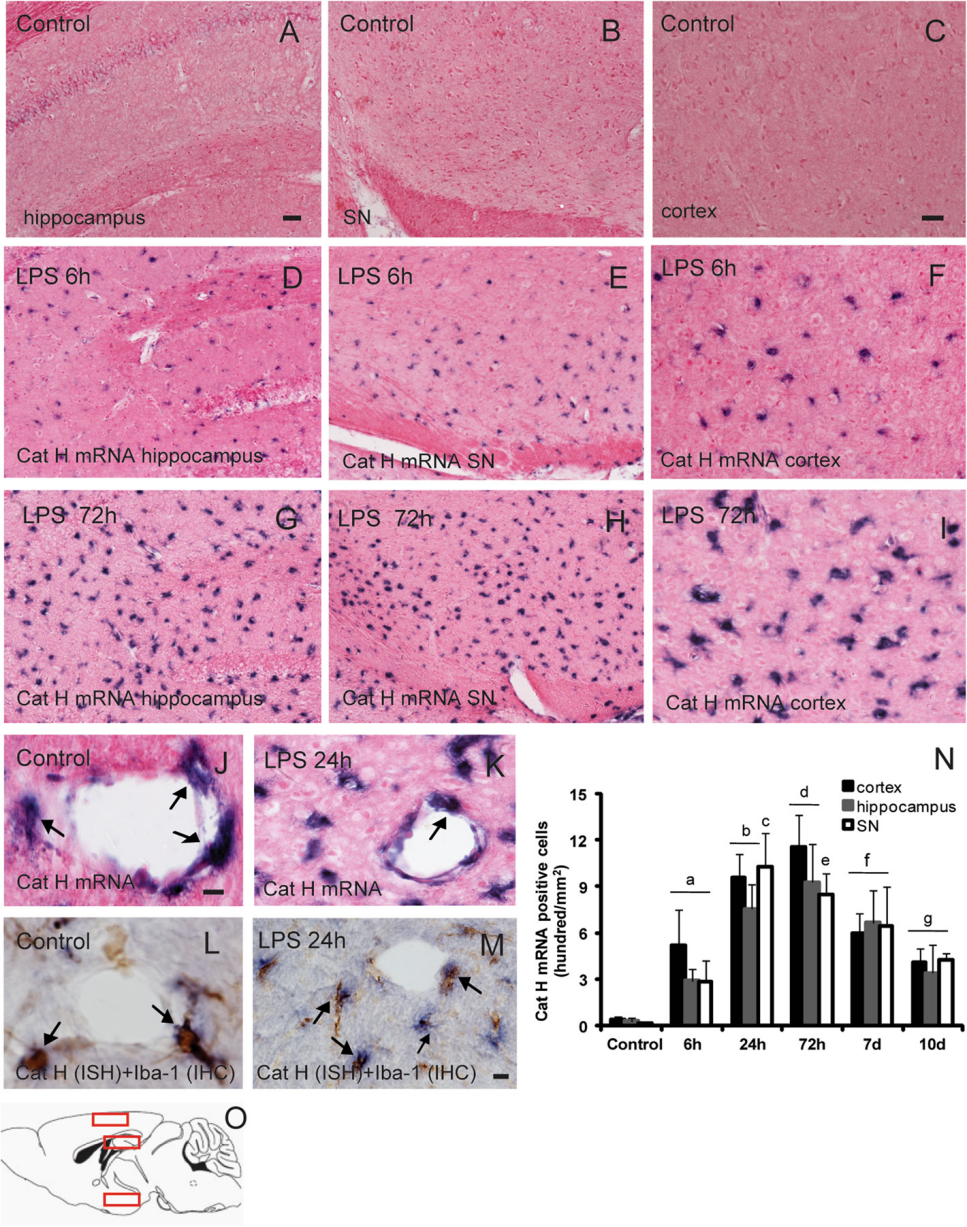
**Microglial Cat H expression was induced at a transcriptional level after LPS injection.** ISH staining showed that at 6 h after LPS injection, Cat H mRNA expression was detected in the hippocampus **(D)**, SNr **(E),** and cerebral cortex **(F)**. At 72 h after LPS injection, the intensity and density of Cat H mRNA positive cells were both increased in the above regions **(G-I)**. In the control, brain Cat H mRNA was absent in the brain **(A-C)**, present only close to the blood vessels (arrows in **J**). Double staining (Cat H ISH following Iba-1 IHC) showed that cellular localization of Cat H mRNA was perivascular microglia (arrows in **L**). At 24 h after LPS injection, Cat H mRNA positive signals appeared in microglia in the brain parenchyma, in addition to perivascular microglia (arrows in **K**, **M**). The quantification results of Cat H mRNA expression in brain regions analyzed after LPS injection were shown in **(N)**. The detected regions were illustrated in sagittal section of the brain **(O)**. Data were expressed as mean + SEM from three independent experiments. ^a^ *P*, ^f^ *P*, ^g^ *P* < 0.05 *vs* control; ^b^ *P* < 0.01 *vs* 6 h; ^c^ *P* < 0.05 *vs* 72 h; ^d^ *P* < 0.05 *vs* 24 h; ^e^ *P* < 0.01 *vs* 6 h. Scale bar = 50 μm (A, B, D, E, G, H); 20 μm (C, F, I); 10 μm (J; K, L, M). *n* = 5.

In this study, we first investigated the Cat H expression at transcriptional level in the normal brain. The ISH results showed that Cat H mRNA was absent in the brain parenchyma, only present in cells with glial morphology, close to the blood vessels (Figure 1A-C,J), also in non-parenchymal sites, such as leptomeninges and the choroid plexus in ventricles.

Next, we investigated the expression pattern of Cat H mRNA in LPS-evoked neuroinflammation. Previously, we reported that a single intraperitoneal injection of LPS (5 mg/kg) on mouse-evoked apparent systemic inflammation and further triggered characterized by activation of Iba-1 positive microglia/macrophages and release of inflammatory mediators (IL-1β, TNF-α, and iNOS) in the brain [18]. Here, we first investigated Cat H expression in neuroinflammation evoked by intraperitoneal injection of LPS at a low dose of 0.1 mg/kg. The ISH staining showed no positive Cat H mRNA signals seen in the brain at 24 h after LPS injection, and real-time quantitative PCR results further confirmed that LPS (0.1 mg/kg, i.p.) failed to induce Cat H mRNA expression in the brain (see Additional file 1). Thus, we next established the mouse neuroinflammatory model with a high dose of LPS (5 mg/kg, i.p.) to investigate the expression patterns of Cat H in the brain.

We especially observed and analyzed brain regions known to be affected in neurodegenerative diseases, such as the cerebral cortex, hippocampus, and SNr. ISH staining showed that at 6 h after LPS injection, Cat H mRNA was expressed in the hippocampus (Figure 1D), SNr (Figure 1E), and cerebral cortex (Figure 1F). At 24 h after LPS injection, the intensity and density of Cat H mRNA positive signals were both increased in a global pattern. In SNr, the number of Cat H mRNA positive cells was maximal at 24 h. In the cerebral cortex and hippocampus, the highest level of Cat H mRNA expression appeared at 72 h (Figure 1G-I,N). These results indicate that in inflammatory condition, the synthesis of Cat H is markedly up-regulated at transcriptional level. Ten days after LPS injection, Cat H mRNA expression was decreased nearly to the level of 6 h for each region investigated (Figure 1N).

Furtherly, we confirmed the cellular localization of Cat H mRNA by double stainings in the following combination: NeuN (neuronal marker, IHC) following Cat H(ISH); Iba-1 (microglia/macrophages marker, IHC) following Cat H (ISH); GFAP (astrocyte marker, IHC) following Cat H (ISH). The results showed that in the normal brain, Cat H mRNA positive cells around vessels were perivascular microglia/macrophages (Figure 1L), confirming the previous studies [48,50-52]. Similarly, after LPS injection, Cat H mRNA was predominantly expressed in the parenchymal and perivascular microglia/macrophages (Figure 1K,M), rarely in astrocytes and neurons.

**Figure 2:**
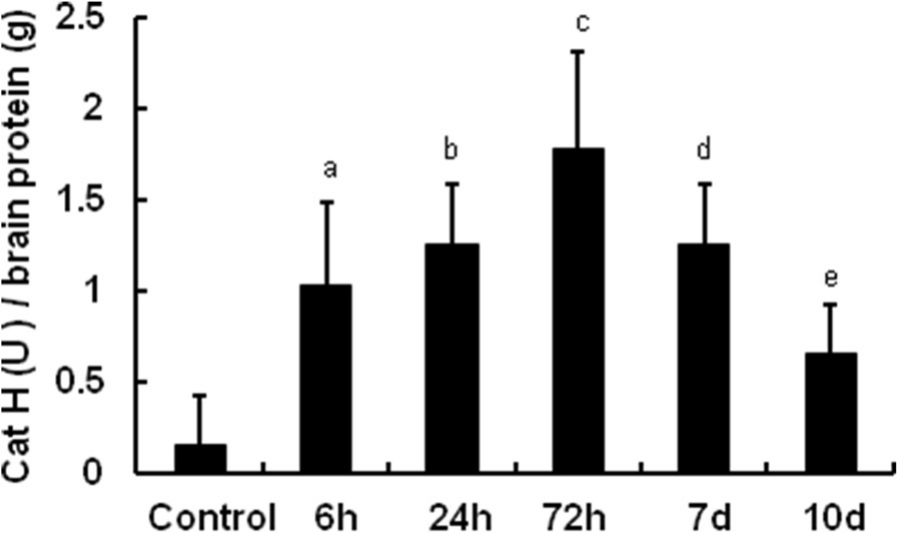
**The expression of Cat H protein was increased in the brain after LPS injection.** ELISA assay showed that brain Cat H protein expression was increased and maximal at 72 h after LPS injection then decreased and maintained at a low level at 10th day. Data were expressed as mean + SEM from three independent experiments. ^a,e^ *P* < 0.05 *vs* control; ^b,d^ *P* < 0.01 *vs* control; ^c^ *P* < 0.05 *vs* b,d. *n* = 5.

Cat H IHC staining is hard to show the spatial expression pattern of Cat H protein appropriately due to its extracellular secretion, therefore ELISA was used instead. The results showed that the level of Cat H protein expression was strikingly increased following LPS challenge with similar alteration tendency of Cat H mRNA (Figure 2). The results indicate that Cat H expression in the brain is regulated at both the transcriptional and translational levels in neuroinflammation.

Taken together, our results indicate that in normal condition, the level of Cat H expression in the brain was low, and LPS-evoked neuroinflammation could induce Cat H expression in microglia.

### Proinflammatory cytokines increased microglial Cat H release and activity *in vitro*

IFN-γ and LPS stimulation can cause a significant increase in the expression and activity of Cat H in macrophages [53,54]. Moreover, Cat H can be secreted via the secretory lysosome system in inflammatory diseases [55-57]. In our study, we wondered whether proinflammatory stimulation can affect Cat H secretion and activity in microglia. We examined Cat H level in culture media of the BV2 cells or primary microglia treated with various concentrations of LPS (10, 100, 1,000 ng/mL), IL-1β, IL-6, TNF-α (for all 1, 10, and 100 ng/mL), and IFN-γ (50, 500, 5,000 ng/mL).

We first determined the nitrite level in the culture media following 24-h treatment with LPS (10 ng/mL), IL-1β, IL-6, TNF-α (for all 1 ng/mL), or IFN-γ (50 ng/mL). There was a significantly higher level of nitrite in the media of BV2 cells and primary microglia after treatments, compared to untreated cells (see Additional file 2). This indicates that the proinflammatory stimuli were sufficient to induce microglial activation, even at the lowest concentrations.

**Figure 3:**
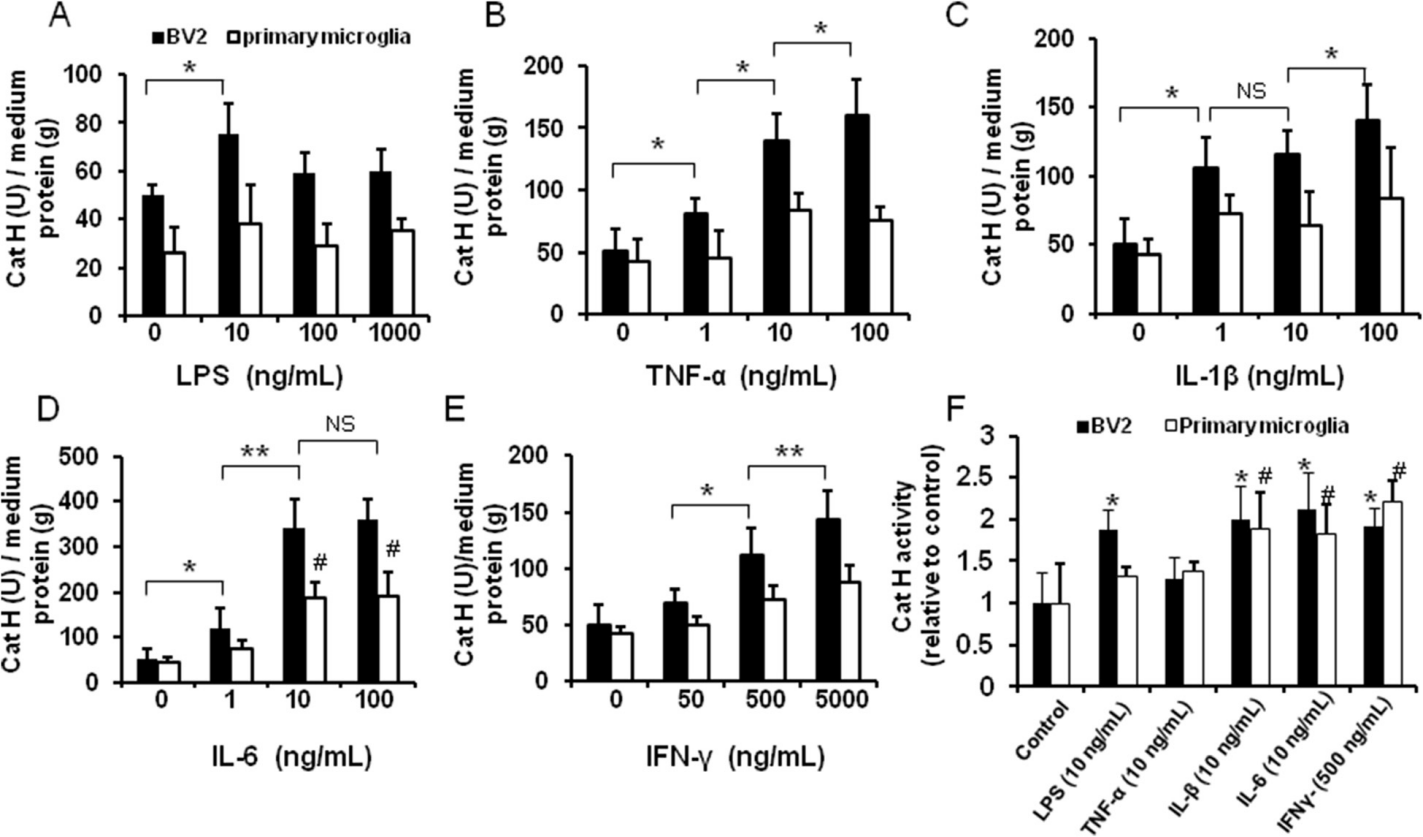
**Proinflammatory cytokines induced microglial Cat H release and up-regulated Cat H activity** ***in vitro.*** The level of Cat H in the media of BV2 cells and primary microglia after various inflammatory treatments for 24 h was measured by ELISA and shown in **(A-E)**. The activity of Cat H in the media of BV-2 cells and primary microglia after various inflammatory treatments was measured and shown in **(F)**. Data were expressed as mean + SEM from three independent experiments. *, #: *P* < 0.05; **: *P* < 0.01, NS: no significance in **(A-E)**, *, #: *P* < 0.05 *vs* control in **(F)**.

Then, we examined the Cat H release in the culture media of BV2 cells or primary microglia by ELISA. We observed the low level of Cat H content in the media in absence of proinflammatory stimuli, suggesting that there is a basal level of Cat H production and secretion in untreated microglia. In contrast, the levels of Cat H were significantly increased in the media of BV2 cells following TNF-α, IL-1β, IL-6, and IFN-γ treatments for 24 h, respectively. Among these, TNF-α and IFN-γ induced Cat H increase in a dose-dependent manner (Figure 3B,E). Besides, IL-6 treatment, to various degrees, induced Cat H release in primary microglia (Figure 3D). However, LPS has little effect on Cat H release in either BV2 or primary microglia (Figure 3A). These results suggest an inducible effect of proinflammatory cytokines on Cat H release in microglia.

Further, we examined Cat H activity in the media of BV2 and primary microglia following 24-hour proinflammatory stimulation. The results showed that stimuli including LPS (10 ng/mL), IL-1β (10 ng/mL), IL-6 (10 ng/mL), and IFN-γ (500 ng/mL) up-regulated Cat H activity significantly, except TNF-α (10 ng/mL), compared to untreated cells (Figure 3F).

These results suggest that proinflammatory stimulation can elevate Cat H activity, which could be associated with increased Cat H secretion.

### Cat H induced microglial release of proinflammatory cytokines which could be attenuated by neutralizing antibody

The previous studies demonstrated that Cat H can provoke acute skin inflammatory response mediated by cytokines and chemokines [40]*.*

In our study, we have found increased expression of proinflammatory cytokines and Cat H in the brain in neuroinflammation. Considering that activated microglia are the main cellular sources of inflammatory mediators in CNS, we raised our hypothesis that Cat H could induce microglial activation and further promote inflammatory mediator release in inflamed CNS.

To testify our hypothesis, we collected the media of BV2 cells incubated with LPS (10 ng/mL) for 24 h, in which the increase of Cat H content was confirmed previously. Then, we transferred the conditioned media to BV2 cells and incubated for another 24 h. To identify the specific effect of Cat H on BV2 cells, in other groups, we additionally added neutralizing Cat H antibody (N18, 1:100 of final volume of media) to BV2 cells. We found that the LPS-treated media promoted the microglial TNF-α, IL-1β, IL-6, and IFN-γ release, but addition of Cat H antibody did not change their release from microglia (data not shown).

**Figure 4:**
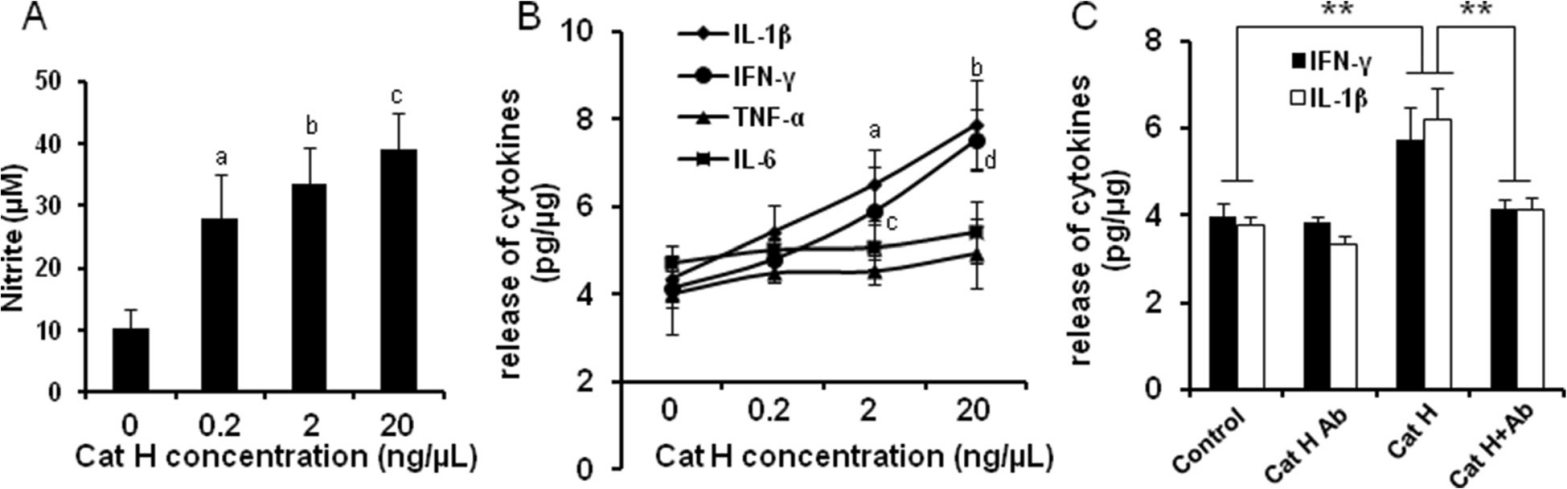
**Cat H induced microglial activation and promoted release of proinflammatory cytokines** ***in vitro.*** The level of nitrite in the media of BV2 cells following various concentrations of Cat H treatments for 24 h was shown in **(A)**. Release of cytokines in the media of BV2 cells treated with various concentrations of Cat H was measured by ELISA and shown in **(B)**. The effect of Cat H antibody on release of cytokines in BV2 cells was shown in **(C)** (Cat H concentration is 2 ng/μL). Data were expressed as mean + SEM from three independent experiments. ^a,b^ *P* < 0.01 *vs* control; ^c^ *P* < 0.05 *vs* a in A; ^a,c^ *P* < 0.05 *vs* control; ^b,d^ *P* < 0.05 *vs* a, c in B. **: *P* < 0.01 in C.

Considering diversity and complexity of inflammatory mediators in the conditioned media of LPS-treated BV2 cells, we added specifically recombinant active Cat H (0.2, 2, 20 ng/μL) to BV2 cells, respectively, to rule out possible influences of other components in the media on BV2 cells. We found that NO production and IL-1β and IFN-γ release were increased dose dependently after addition of Cat H, while TNF-α and IL-6 release was not affected significantly(Figure 4A,B). Furthermore, we found that the release of L-1β and IFN-γ was decreased significantly following combined addition of Cat H (2 ng/μL) and neutralizing antibody (N18, 1:100 of final volume of media) for 24 h (Figure 4C). These results support our hypothesis that Cat H could induce microglial activation and promote subsequent release of proinflammatory cytokines.

Taken together, proinflammatory cytokines induced microglial Cat H release and vice versa, which suggests that there could be interaction between Cat H and proinflammatory cytokines mediated by activated microglia.

### Cat H induced neuronal death *in vitro*

Cathepsins can cause neuronal apoptosis in neurodegeneration [58-63], but so far, it is unknown whether Cat H is toxic to neurons in neuroinflammation. Next, we observed the influence of Cat H on neuronal death by adding active Cat H protein (2, 20 ng/μL) into the culture media of Neuro2a for 24 h and measuring annexin-V positive cells (A^+^/PI^−^) and double stained cells (A^+/^PI^+^) representing apoptotic and necrotic (or late apoptotic) cells, respectively, by flow cytometer.

**Figure 5:**
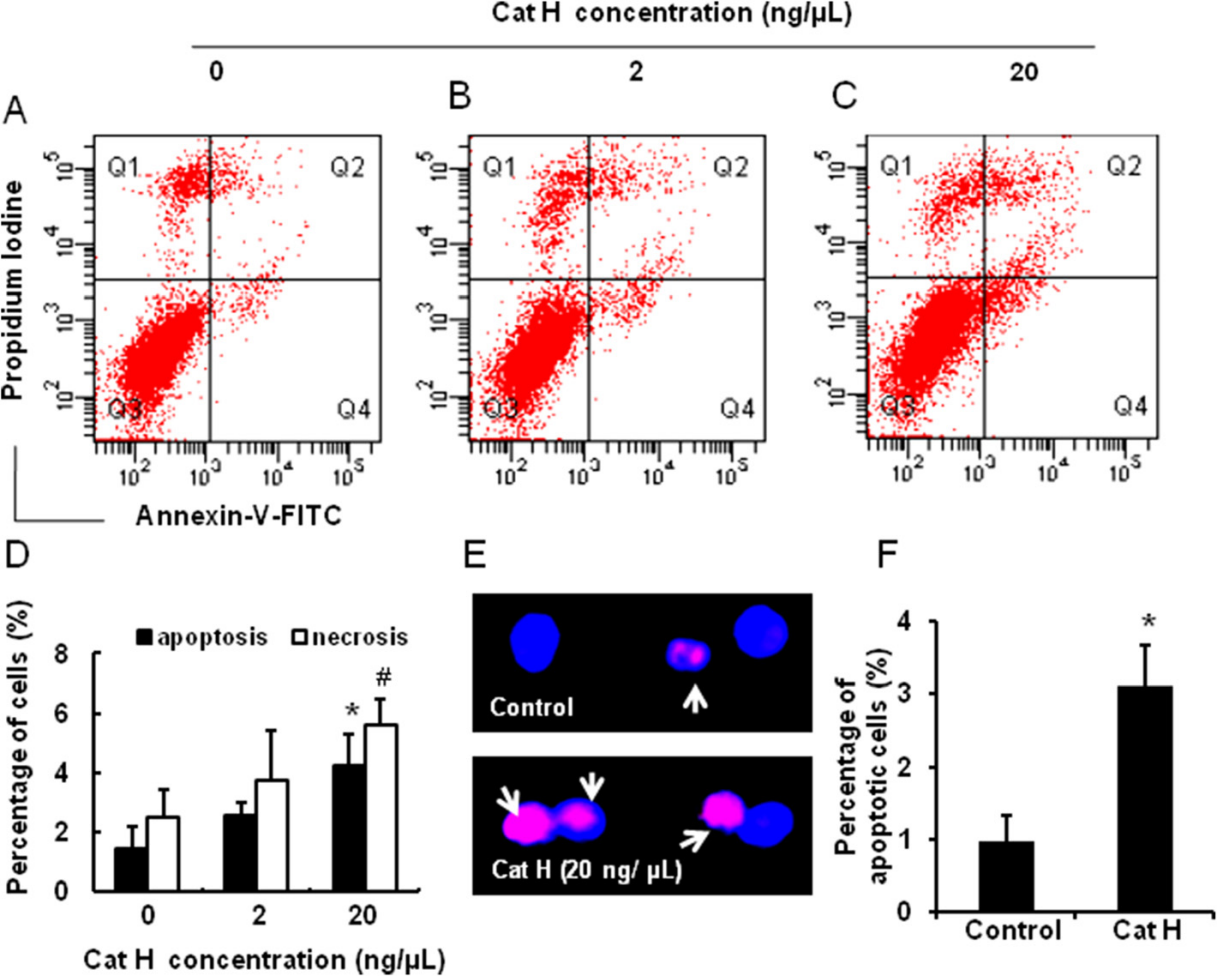
**Cat H induced neuronal death** ***in vitro.*** The effect of Cat H in various concentrations on Neuro2a cell survival was measured by flow cytometry **(A-D)**; TUNEL positive staining exhibited the morphological changes of Neuro2a cells after addition of Cat H **(E-F)**. Data were expressed as mean + SEM from three independent experiments. *, #: *P* < 0.05 *vs* control.

As shown in Figure 5A-D, both percentages of apoptosis and necrosis in Cat H (20 ng/μL)-treated cells were significantly higher than those of control cells, indicating that Cat H could have a neurotoxic influence on neurons resulting in neuronal death.

Further, TUNEL positive staining in *in situ* detection exhibited apoptotic Neuro2a cells with condensed nuclear morphology following exposure to Cat H (20 ng/μL) for 24 h (Figure 5E). A significant increase of apoptotic cells was found following Cat H treatment, compared to untreated cells (Figure 5F).

These results suggest that Cat H could induce neuronal death through exerting neurotoxicity on neurons, which may play a crucial role in maintenance and aggravation of neuroinflammatory diseases.

## Discussion

An inflammatory reaction in the brain is primarily characterized by activation of parenchymal microglial cells. Microglia-mediated neuroinflammation is thought to promote brain damage in various neurodegenerative disorders [8-12].

Systemic injection of LPS is often used in experimental models of neuroinflammation, for example, Parkinson’s disease models [46,47]. In our previous study, LPS (5 mg/kg, i.p.) injection resulted in microglial activation and promoted production of proinflammatory cytokines in the mouse brain. Qin *et al.* reported that inflammatory responses evoked by LPS (5 mg/kg, i.p.) injection could persist for several months in the brain [46,47]. In this study, we found that LPS systemic injection (5 mg/kg) markedly up-regulated the level of Cat H expression predominantly in activated microglia. A low dose of LPS (0.1 mg/kg, i.p.) injection in our pre-experiments evoked neuroinflammation but failed to induce brain Cat H expression.

Our *in vitro* study further demonstrated that the proinflammatory cytokines (TNF-α, IL-1β, IL-6, IFN-γ) have an inducible effect on release and enzymatic activity of Cat H in microglia. The gradient doses of proinflammatory agents for stimulating microglia were derived from literatures concerned [64,65]. It is notable that Cat H level in the culture medium of primary microglia was lower than BV2 cells following inflammatory stimuli. This difference may be associated with biological characteristics of two types of cells. Additionally, the frequency of propagation of BV2 cells may contribute to the different biological characteristics from primary microglia.

**Figure 6:**
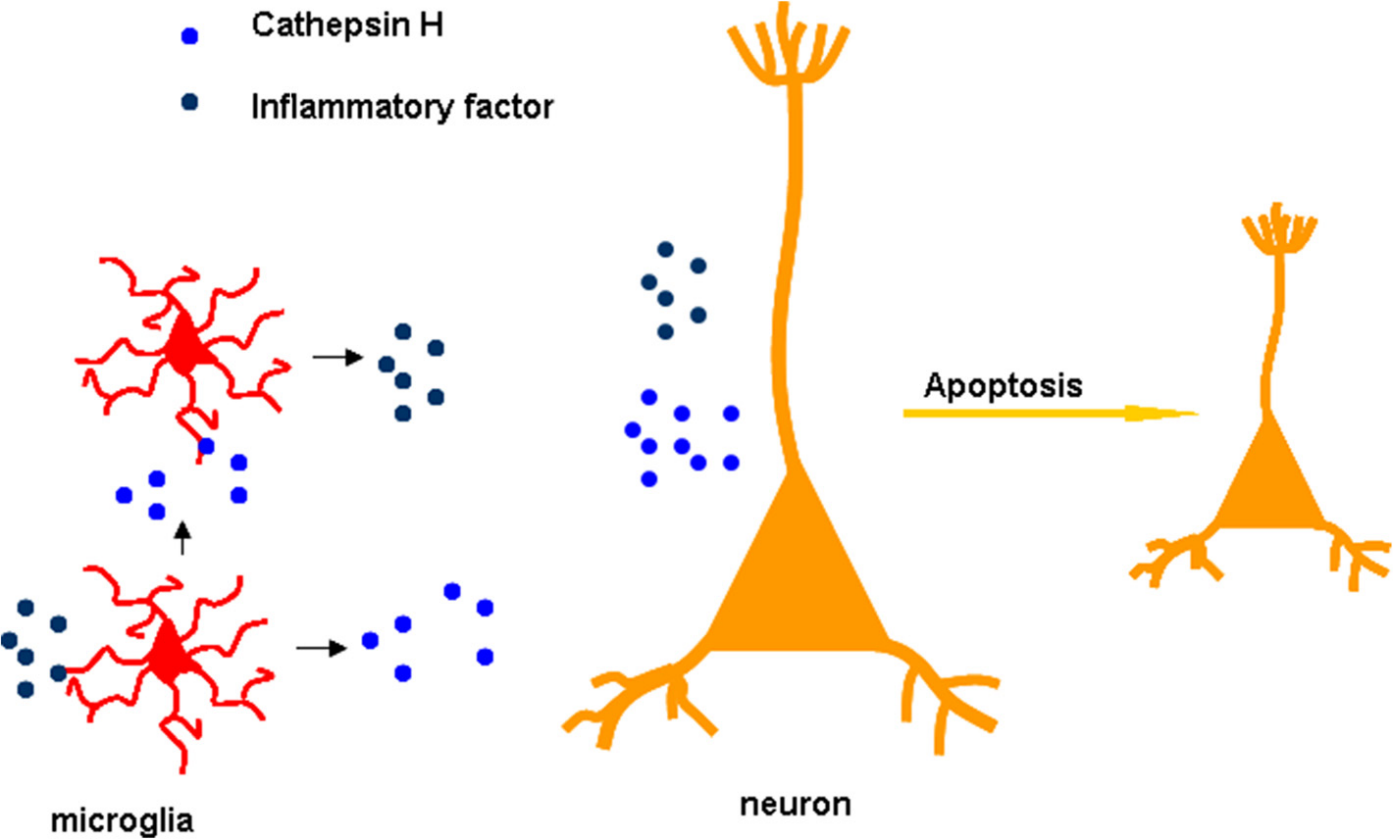
**The function link of Cat H with microglia activation inducing neuronal apoptosis.** Inflammatory cytokines induced Cat H release from microglia and enhanced its activity. Also, Cat H induced microglial activation characterized by the release of inflammatory cytokines. Cat H, alone or in combination of inflammatory cytokines, contributes to neuronal apoptosis.

On the other hand, active Cat H induced microglial activation characterized by the release of NO and various proinflammatory cytokines. More importantly, active Cat H was toxic to neurons and induced neuronal death. These findings lead us to a hypothesis that the functional link of Cat H with microglia activation might involve a self-sustaining amplifying circle, contributing to the initiation and maintenance of microglia-driven chronic inflammation (Figure 6). To the best of our knowledge, this is the first report to reveal the regional expression and cellular localization of Cat H in the brain and highlight its potentially functional role in neuroinflammation.

Several clinical reports showed that Cat H was involved in pathological processes of many peripheral inflammatory diseases, in which the levels of Cat H were dramatically increased in the secretory compartment of cells or in the serum of patients [42,66]. These findings indicate that Cat H can be secreted via the secretory lysosome system during pathological processes. The secretive property of Cat H would allow access to the extracellular environment and exert the extracellular action [67].

Considering the antibody for Cat H, immunoassay used in our study is specific for mature single-chain form of Cat H (28 kDa); therefore, we rationally speculate that Cat H protein detected in media of microglia following the inflammatory activation was in mature form, not in inactive form. The results confirm the previous reports that extracellularly secreted Cat H is mainly in mature form, unlike cathepsins B, K, and S which were mostly zymogens [32,42].

Mature Cat H has unique aminopeptidase activity, which is distinguished from other cysteine proteases, such as cathepsins B and L [68]. The crystal structure of mature Cat H determines greatly its limited endopeptidase activity on synthetic and natural substrates [35,39]. Aminopeptidases are exopeptidases that catalyze the hydrolysis of amino acid residues from the N-terminus of peptides or proteins. Disturbance of the normal balance of aminopeptidase activity may lead to inflammatory events and tumor progression [36-38]. As demonstrated in our enzyme assay, the aminopeptidase activity of Cat H is increased in the media of microglia following inflammatory stimulation. It is known that the cathepsin activity is regulated in many ways, that is, gene expression, zymogen activation, cellular environment, and presence or absence of inhibitors [54]. In this study, the increase of Cat H enzymatic activity could be due, in part, to the expression level of the fully processed mature Cat H form. However, it is hard to explain the differences between protein and activity of Cat H in primary microglia. We speculate that Cat H activity may be associated with other factors, apart from Cat H protein content. The mechanisms of regulation of Cat H activity need further study.

To date, the mechanisms of overproduced Cat H involved in immune responses remain unclear. Perez *et al.* found that rats injected with Cat H intracutaneously had acute dermal inflammation characterized by the local accumulation of PMN under the action of chemotactic factors [40]. In our *in vitro* study, recombinant active Cat H protein exerted an inducible impact on the release of IL-1β and IFN-γ in microglia, which could be prevented by specific neutralizing antibody obviously. It implies strongly that Cat H could participate in and even aggravate inflammatory responses through inducing the release of inflammatory factors.

In addition, several studies have identified that Cat H primarily participates in pathological apoptosis, for instance, Nitatori *et al.* reported that Cat H was involved in apoptosis of CA1 pyramidal neurons after brief ischemia [69]. Our *in vitro* study demonstrated that recombinant active Cat H caused significant neuronal death when added directly to the neuroblastoma cell line Neuro2a, indicating that Cat H exerted cytotoxic effects on neurons. The mechanisms of Cat H mediating neuronal death is thought to be associated with cathepsin C. Under certain proinflammatory conditions, Cat H cooperating with cathepsin C can converse pro-granzyme B (proGrB) into granzyme B (GrB) in immune cells including cytotoxic lymphocytes (CTLs), natural killer (NK) cells, activated macrophages, and neutrophils*.* Once released, GrB can cleave extracellular matrix resulting in local proteolysis and tissue damage or further delivered into target cells by pore-forming protein perforin to induce cell death [70,71]. In this study, we cannot ascertain the mechanisms underlying Cat H-mediated neuronal death. We speculate that secreted Cat H may function as a ligand rather than a catabolic enzyme, directly binding to as yet unidentified specific receptors on the surfaces of neurons, which is responsible for triggering intracellular death-related signaling pathways.

## Conclusions

In summary, in the present study, we investigated the temporal and spatial expression patterns of Cat H in the brain in LPS-evoked neuroinflammation. We found the prominent up-regulation of Cat H expression in brain microglia after LPS injection. *In vitro* study further confirmed that proinflammatory cytokines could induce release of Cat H in microglia, which could exert a profound and detrimental impact on microglial activation and neuronal survival. Elucidation of the precise mechanism underlying the neurotoxicity of microglial Cat H may provide insights into how microglia influences neuronal functions and status in neuroinflammation. In addition, control of inflammatory response and inhibition of the Cat H level in the brain would be important for prevention and treatment of neurodegenerative diseases that are associated with excessive microglial activation and subsequent neurotoxic inflammation.

## Additional files

Additional file 1:
**LPS (0.1 mg/kg, i.p.) failed to induce Cat H mRNA expression in the brain at 24 h after injection LPS (5 mg/kg, i.p.) significantly induced Cat H expression.** Cat H mRNA level was analyzed by real-time quantitative PCR. Data were expressed as mean + SEM from three independent experiments. ^a^ *P* > 0.05 *vs* control, ^b^ *P* < 0.01 *vs* control, or a (*n* = 5). Additional file 2:
**LPS or proinflammatory cytokines induced production of NO in the media of BV2 cells and primary microglia.** Data were expressed as mean + SEM from three independent experiments. ^*^ *P* < 0.01, ^#^ *P* < 0.01 *vs* control. ^**^ *P* < 0.001, ^##^ *P* < 0.001 *vs* control.

## Abbreviations

BCIP: 5-bromo-4-chloro-3-indolyl-phosphate
Cat H: cathepsin H
CNS: central nervous system
DIG: digoxigenin; DPPI
DMEM: Dulbecco’s modified eagle medium
ELISA: enzyme-linked immunosorbent assay
FBS: fetal bovine serum
IFN-γ: interferon-γ
IHC: immunohistochemistry
IL: interleukin
iNOS: inducible nitrite oxide synthase
i.p.: intraperitoneal
ISH: *in situ* hybridization
kDa: kiloDaltons
LPS: lipopolysaccharide
mAb: monoclonal antibody
NBT: 4-nitro blue tetrazolium chloride
NO: nitrite oxide
PBS: phosphate-buffered saline
SEM: standard error of the mean
SNr: substantia nigra reticular
TNF-α: tumor necrosis factor-α
