# Supplementary figures and images for: The induction of neuronal death by up-regulated microglial cathepsin H in LPS-induced neuroinflammation
Source: J Neuroinflammation. 2015 Mar 19;12:54. doi: 10.1186/s12974-015-0268-x (PMC4379721; doi:10.1186/s12974-015-0268-x)

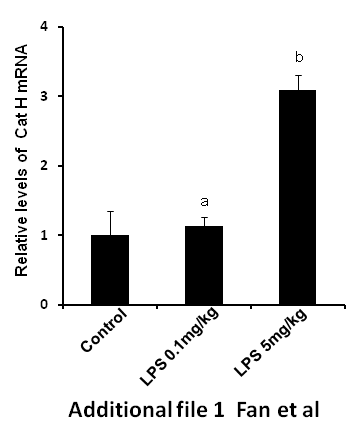

Supplement: Additional file 1: — LPS (0.1 mg/kg, i.p.) failed to induce Cat H mRNA expression in the brain at 24 h after injection LPS (5 mg/kg, i.p.) significantly induced Cat H expression. Cat H mRNA level was analyzed by real-time quantitative PCR. Data were expressed as mean + SEM from three independent experiments. a P > 0.05 vs control, b P < 0.01 vs control, or a (n = 5). [file 12974_2015_268_MOESM1_ESM.tiff]

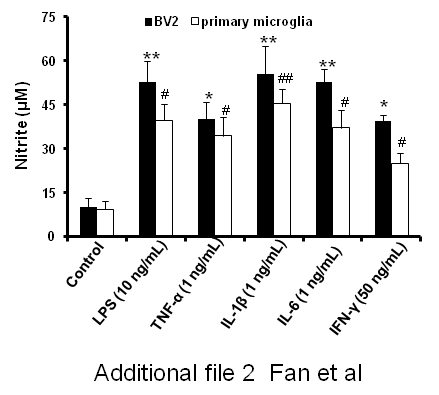

Supplement: Additional file 2: — LPS or proinflammatory cytokines induced production of NO in the media of BV2 cells and primary microglia. Data were expressed as mean + SEM from three independent experiments. * P < 0.01, # P < 0.01 vs control. ** P < 0.001, ## P < 0.001 vs control. [file 12974_2015_268_MOESM2_ESM.tiff]
